# Changes in sleep and the prevalence of probable insomnia in undergraduate university students over the course of the COVID-19 pandemic: findings from the U-Flourish cohort study

**DOI:** 10.1192/bjo.2023.597

**Published:** 2023-11-07

**Authors:** Nathan King, William Pickett, Charles D. G. Keown-Stoneman, Christopher B. Miller, Melanie Li, Anne Duffy

**Affiliations:** Department of Public Health Sciences, Queen's University, Canada; Department of Public Health Sciences, Queen's University, Canada; and Department of Health Sciences, Brock University, Canada; Dalla Lana School of Public Health, University of Toronto, Canada; and Li Ka Shing Knowledge Institute, St Michael's Hospital, Toronto, Canada; Big Health Inc., San Francisco, USA; and Sir Jules Thorn Sleep and Circadian Neuroscience Institute (SCNi), Nuffield Department of Clinical Neurosciences, University of Oxford, UK; Department of Biology, Queen's University, Canada; Department of Psychiatry, Queen's University, Canada; and Department of Psychiatry, University of Oxford, UK

**Keywords:** Sleep–wake disorders, epidemiology, university students, COVID-19 restrictions, health promotion and early intervention

## Abstract

**Background:**

Sleep problems associated with poor mental health and academic outcomes may have been exacerbated by the COVID-19 pandemic.

**Aims:**

To describe sleep in undergraduate students during the COVID-19 pandemic.

**Method:**

This longitudinal analysis included data from 9523 students over 4 years (2018–2022), associated with different pandemic phases. Students completed a biannual survey assessing risk factors, mental health symptoms and lifestyle, using validated measures. Sleep was assessed with the Sleep Condition Indicator (SCI-8). Propensity weights and multivariable log-binomial regressions were used to compare sleep in four successive first-year cohorts. Linear mixed-effects models were used to examine changes in sleep over academic semesters and years.

**Results:**

There was an overall decrease in average SCI-8 scores, indicating worsening sleep across academic years (average change −0.42 per year; *P*-trend < 0.001), and an increase in probable insomnia at university entry (range 18.1–29.7%; *P*-trend < 0.001) before and up to the peak of the pandemic. Sleep improved somewhat in autumn 2021, when restrictions loosened. Students commonly reported daytime sleep problems, including mood, energy, relationships (36–48%) and concentration, productivity, and daytime sleepiness (54–66%). There was a consistent pattern of worsening sleep over the academic year. Probable insomnia was associated with increased cannabis use and passive screen time, and reduced recreation and exercise.

**Conclusions:**

Sleep difficulties are common and persistent in students, were amplified by the pandemic and worsen over the academic year. Given the importance of sleep for well-being and academic success, a preventive focus on sleep hygiene, healthy lifestyle and low-intensity sleep interventions seems justified.

## Background

In the transition to university, students assume increased responsibility for managing their schedules, lifestyle and sleep habits. Heightened stress related to academic pressures, making new friends, living away from home and increased substance use can affect sleep patterns and contribute to poor sleep quality in post-secondary students.^1^ In 2018, approximately 18% of first-year undergraduates at Queen's University, Canada (12% of men and 20% of women) reported sleep difficulties indicative of probable insomnia at school entry.^2^ Insomnia in university students is associated with reduced academic performance,^3,4^ and common mental health concerns such as anxiety and depression.^5,6^

The COVID-19 pandemic had a particularly disruptive and generally negative effect on university student well-being and mental health.^7,8^ Shifts to remote learning, changes in the frequency and mode of examinations, increased uncertainty about current and future prospects, and social isolation from peers have been associated with increased levels of stress and anxiety.^7,8^ During the peak of pandemic-related restrictions, remote learning and asynchronous lectures put more responsibility on students to regulate their schedules and study habits, not uncommonly under non-ideal learning conditions. Therefore, studying under pandemic conditions may have further increased the risk of sleep difficulties in university students.

A large international survey study including over 25 000 adults across 14 countries reported a 10% or more increase in sleep difficulties (e.g. onset and maintenance problems) and related daytime problems (i.e. fatigue and daytime sleepiness) during the COVID-19 pandemic from May to August 2020. Social restrictions and financial concerns were significantly associated with reported sleep difficulties. Although sleep worsening was reported across all countries, poor sleep quality was particularly high in this study in Canada and the UK (four-fold and three-fold increases, respectively). It was speculated that this may reflect a pandemic stage effect, in that more sleep problems were reported in countries where the pandemic and related public health restrictions were escalating compared with those where it was more stable.^9^

## Present study

The U-Flourish Student Well-Being Survey study launched in 2018 at Queen's University in Canada.^2^ This longitudinal study engaged robust cohorts of first-year undergraduate students to complete biannual electronic surveys at entry and completion of each academic year. Therefore, we had a unique opportunity to describe changes in sleep over varying stages of the COVID-19 pandemic from the autumn of 2018 (pre-pandemic) to the spring of 2022 (return to in-person classes and relaxed pandemic restrictions). We hypothesised that sleep difficulties would be common at entry to university, increase over the academic year and be most pronounced during the peak of the pandemic (associated with remote learning and the most severe pandemic-related restrictions). Our specific study objectives were to (a) describe sleep in successive student cohorts at entry to university over different stages of the pandemic, (b) examine changes in student sleep over the academic year and different stages of the pandemic, and (c) explore associations between modifiable risk factors and screening positive for probable insomnia. To our knowledge, this is the first large-scale study to describe sleep patterns in undergraduate university students, including documentation of trends in sleep over the course of the COVID-19 pandemic.

## Method

### Data source

Data from this study come from four consecutive cohorts of the longitudinal U-Flourish Student Well-being and Academic Success Survey study described in detail elsewhere.^2,10^ Since the autumn of 2018, all first-year undergraduates entering Queen's University have been invited to complete online surveys at school entry (mid-September) and the end of the academic year (mid-March), before to winter examinations. Follow-up surveys were repeated at the same time points each successive year. The survey collects data on demographics, mental health risk factors, current lifestyle and indicators of mental health and well-being, using validated measures. Participation incentives and a student-led engagement campaign that included in-class presentations, social media posts and the use of working booths at university events and in campus residence buildings, were used to encourage student participation. Before the COVID-19 pandemic, the baseline response rates for cohort 1 (*n* = 3029) and cohort 2 (*n* = 2949) were 58%. During the pandemic, when in-person engagement activities were not possible, the response rate dropped to 23.3% (cohort 3, *n* = 1472). In cohort 4, students started the year in-person and the response rate was 35% (*n* = 1991). The response rates on the first spring follow-up survey by cohort were 64.3%, 37.4%, 33.2% and 41.1%, respectively (Supplementary Fig. 1 available at https://doi.org/10.1192/bjo.2023.597**)**. All students provided informed consent to access and complete the survey.^10^

### Study variables

#### Year of study

To describe sleep in students beginning university over the course of the pandemic, we compared the sleep of four successive cohorts of first-year students at school entry (autumn 2018 to autumn 2021). To examine changes in sleep over the academic year and throughout the pandemic, we combined cohorts, using all available data to compare sleep across academic semesters and years. Each autumn term and academic year corresponded to a unique stage of the pandemic, as described below.

##### Pre-pandemic conditions (cohort 1, 2018–2019)

Students entered university in the autumn of 2018 and completed their first academic year in the spring of 2019, under normal pre-pandemic conditions.

##### Transitional conditions (cohort 2, 2019–2020)

Students entered university in the autumn of 2019, under normal pre-pandemic conditions. The World Health Organization declared COVID-19 a pandemic on 11 March 2020, and Queen's University suspended undergraduate classes on 16 March before moving online on 23 March. The U-Flourish Spring survey was sent out on the first day courses moved online, and was therefore completed during a time of great uncertainty when the full impact of the pandemic was not yet realised.

##### Peak pandemic conditions (cohort 3, 2020–2021)

Students started their undergraduate studies online. In the autumn of 2020, Queen's University campus was closed and the 2020–2021 academic programming was completed fully online, under pandemic-related public health restrictions such as social distancing and business and recreational facility closures. During this time, Queen's University housing residences operated at half capacity.

##### Hybrid pandemic conditions (cohort 4, 2021–2022)

Students started the 2021–2022 academic year on campus with the following pandemic restrictions in place: mandatory masking, self-screening, and vaccination and social distancing safety protocols. The year was a hybrid of in-person and online learning, as the university reacted to the evolving pandemic situation and responded to government recommendations. It started in person, but December examinations were moved online in response to the Omicron variant, and courses remained online until the end of February. Residences were operating at close to full capacity (93%), offering only single-room accommodations.

#### Demographic characteristics

Age in years, international status and gender identity were self-reported. Students identified their ethnicity and reported personal and family history (first-degree relatives) of a diagnosed mental disorder from standard lists. The highest level of education completed by either parent was recorded. As possible risk factors for sleep disorders, experiences of childhood physical and/or sexual abuse, and being bullied or teased by peers were measured with items from the Childhood Experience of Care and Abuse questionnaire.^11^ Symptoms of anxiety and depression at university entry were measured with the Generalised Anxiety Disorder 7 scale^12^ and the Patient Health Questionnaire 9,^13^ respectively. On both scales, a score of 10 or more indicates a positive result for clinically significant symptoms.

#### Sleep variables

The eight-item Sleep Condition Indicator (SCI-8) was used to assess sleep and identify screen positives for probable insomnia. Higher total scores (range 0–32) indicate better sleep, and a screening cut-off of ≤16 is used to identify students with probable insomnia.^14,15^ To describe the nature of sleep difficulties and provide context for the total SCI-8 score, individual items of the SCI-8 were dichotomised by applying cut-offs that were informed by the scoring guidelines for the scale.^14^ A more strict cut-off was used for three of the eight items based on the distribution of responses in our sample.

#### Lifestyle variables

As possible correlates of sleep disorders, frequency of binge drinking, defined as consuming five or more drinks on one occasion, and cannabis use during the past month were selected from a set of options provided. Exercise frequency in the past month was measured with the item ‘How often have you worked out, gone to the gym, or attended a fitness class?’. Students also reported how often in the past month they set aside time for self-care or recreational activities (i.e. to relax, recharge, do something you enjoy, etc.), and how much time they usually spent per day engaged in passive screen time unrelated to their studies (i.e. watching television or videos or using an application or game).

### Analysis

We used SAS version 9.4 for Windows software to conduct the analyses for describing sleep in successive cohorts over different stages of the pandemic and exploring associations between risk factors and positive insomnia screening. We used R version 4.1.3 for Windows software (R Foundation, Vienna, Austria; see https://cran.r-project.org/bin/windows/base/) to conduct the analysis for changes in student sleep over the academic year and during the pandemic.

To account for differential response rates across the baseline surveys, an inverse probability weighting approach was used.^16^ Student cohorts 1, 3 and 4 were weighted to cohort 2 (the largest sample), using the following risk factors for sleep problems: age, gender, international status, personal and family history of a mental disorder, history of childhood physical or sexual abuse, history of childhood bullying and parental education. Sleep difficulties and probable insomnia at school entry were described by cohort, using weighted proportions and 95% confidence intervals. We similarly described positive screening for probable insomnia in male and female students (there was insufficient power to stratify by non-binary gender identity).

Multivariable log-binomial regression models were used to examine associations between cohort membership and the likelihood of sleep difficulties at school entry. To capture changes in risk over time, separate models were run comparing each successive cohort to the previous one. These associations were not significantly different in male and female students (all *P*-interaction > 0.10), so only results from the full sample are presented. The proportion of students missing data on key covariates and/or the SCI-8 at baseline ranged from 12.6% (cohort 2) to 24.7% (cohort 4).

Using longitudinal data from all cohorts, changes in average sleep quality (SCI-8) were examined across semesters and stages of the pandemic. First, a minimally adjusted linear mixed-effects model (LMM) was fitted, accounting for semester (survey) to assess potential non-linear patterns of mean sleep quality scores over time (Supplementary Fig. 2). Next, a full LMM was fitted, adjusting for demographic characteristics, cohort membership and categorical semester, as there was visual evidence of non-linearity. This model included interaction terms between cohort and semester, allowing each cohort to have their own mean longitudinal trajectory, as well as random intercepts and slopes for semester number to account for lack of independence between within-participant measurements. Average sleep scores were estimated for semesters and academic years with the fully adjusted LMM, and contrasts were performed to compare mean sleep scores in the spring and autumn semester of each academic year, and successive academic years representing different stages of the pandemic.^17^ Ten multivariate multiple imputed data-sets were used for missing participant-specific covariate data,^18^ and inverse probability weighting was used to account for potential bias resulting from loss to follow-up.^19^ Results were pooled with Rubin's rules.^18^

Bivariate and multivariable log-binomial regression models were used to examine the association between demographic and theoretically important risk factors measured at the beginning of the academic year and probable insomnia at completion of the year. This analysis was conducted with data from all four cohorts collected during the 2021–2022 academic year, which most closely represented the current situation. The Markov chain Monte Carlo method of multiple imputation was used to impute missing data with effect estimates generated based on ten imputed data-sets, using all available data (*N* = 3434).^20^ The imputation model included demographic characteristics, theoretically important risk factors and clinically significant insomnia symptoms at baseline and follow-up.

### Ethics

The authors assert that all procedures contributing to this work comply with the ethical standards of the relevant national and institutional committees on human experimentation and with the Helsinki Declaration of 1975, as revised in 2008. All procedures involving human participants were approved by the Queen's University and Affiliated Teaching Hospitals Research Ethics Board (approval number HSREB PSIY-609–18).

## Results

The four incoming cohorts of students surveyed in successive autumn terms are described in Table 1. The majority of students in cohort 2 (the reference cohort) self-identified as female (68%) and of White ethnicity (67%). Approximately 9% were international students, and the most common programmes of study were arts, humanities and social sciences (34%), life and physical sciences (28%), and engineering and applied science (17%). Nearly a third of students indicated a lifetime history of a mental disorder, and almost half (48%) reported a family history of a mental disorder.

**Table 1:** Description of four successive U-Flourish cohorts at university entry

	Cohort 1 autumn 2018, pre-pandemic		Cohort 2 autumn 2019, transitional pandemic		Cohort 3 autumn 2020, peak pandemic		Cohort 4 autumn 2021, hybrid pandemic	
	*n*	(%)	*n*	(%)	*n*	(%)	*n*	(%)
Overall	2501	(100)	2575	(100)	1201	(100)	1500	(100)
Age, years, mean (s.d.)^a^	18.2	(2.0)	18.0	(1.1)	18.7	(2.5)	18.7	(2.1)
Gender^a^								
Female	1701	(68.0)	1754	(68.1)	808	(67.3)	1092	(72.8)
Male	782	(31.3)	800	(31.1)	377	(31.4)	378	(25.2)
Non-binary	18	(0.7)	21	(0.8)	16	(1.3)	30	(2.0)
Ethnicity								
White	1684	(67.4)	1710	(66.5)	726	(60.5)	928	(61.9)
Asian	490	(19.6)	434	(16.9)	271	(22.6)	297	(19.8)
Black	35	(1.4)	37	(1.4)	22	(1.8)	26	(1.7)
Indigenous	4	(0.2)	6	(0.2)	5	(0.4)	6	(0.4)
Other	35	(1.4)	75	(2.9)	43	(3.6)	65	(4.3)
Multiple	251	(10.0)	311	(12.1)	134	(11.2)	177	(11.8)
Missing	*2*		2		0		1	
Programme of study								
Arts, humanities and social sciences	866	(34.6)	885	(34.4)	451	(37.6)	530	(35.4)
Life and physical sciences	722	(28.9)	729	(28.3)	247	(20.6)	387	(25.8)
Engineering and applied science	391	(15.6)	438	(17.0)	199	(16.6)	158	(10.5)
Business	280	(11.2)	249	(9.7)	77	(6.4)	103	(6.9)
Computing	86	(3.4)	54	(3.4)	43	(3.6)	37	(2.5)
Health sciences	/	/	86	(3.3)	77	(6.4)	126	(8.4)
Other Programmes^b^	156	(6.3)	134	(3.9)	107	(8.9)	158	(10.6)
International student, yes^a^	236	(9.4)	226	(8.8)	113	(9.4)	78	(5.2)
Childhood physical or sexual abuse, yes^a^	559	(22.4)	473	(18.4)	303	(25.2)	467	(31.1)
Childhood bullying, yes^a^	540	(21.6)	511	(19.8)	247	(20.6)	407	(27.1)
Lifetime history of mental disorder, yes^a^	679	(27.2)	787	(30.6)	393	(32.7)	548	(36.5)
Screen positive for anxiety, yes^c^	816	(32.8)	794	(31.1)	474	(39.5)	675	(45.1)
Screen positive for depression, yes^d^	694	(27.9)	766	(29.9)	448	(37.3)	635	(42.4)
Family history of mental disorder, yes^a^	1076	(43.0)	1227	(47.7)	635	(52.9)	823	(54.9)
Parental education, highest completed^a^								
Professional school or doctorate	595	(23.8)	617	(24.0)	250	(20.8)	263	(17.5)
Master's degree	599	(24.0)	572	(22.2)	237	(19.7)	297	(19.8)
Bachelor's or trades/apprenticeship	977	(39.1)	1115	(43.3)	546	(45.5)	687	(45.8)
Completed high School or less	330	(13.2)	271	(10.5)	168	(14.0)	253	(16.9)

Cohorts 1, 3 and 4 were weighted to cohort 2 for the descriptive comparison of sleep patterns.

a. Indicates a variable that was included in the weighting procedure.

b. Other programmes of study include nursing, law and medicine.

c. Screened positive for anxiety at university entry (Generalised Anxiety Disorder 7 score ≥10).

d. Screened positive for depression at university entry (Patient Health Questionnaire 9 score ≥10).

### Description of sleep at university entry over the pandemic

**Fig. 1:**
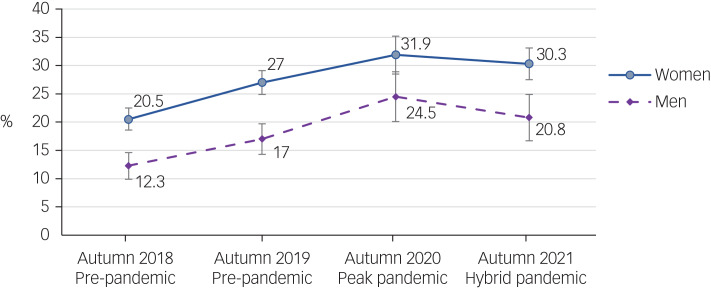
Positive screening rates for probable insomnia (SCI-8 total score ≤16) in first-year students at entry to university from 2018 to 2021, by self-identified gender. SCI-8, Sleep Condition Indicator.

Sleep difficulties in the four successive first-year student cohorts are described in Table 2. The proportion of students screening positive for probable insomnia increased from 18% in cohort 1 (autumn 2018) to 30% in cohort 3 (autumn 2020) (*P*-trend < 0.001), followed by 28% in cohort 4 (autumn 2021). Approximately a fifth of students reported poor or very poor sleep quality, which worsened during the peak of the pandemic (autumn 2020). The extent to which sleep reportedly affected mood, energy and relationships varied over the cohorts and ranged from 36 to 48%, whereas about half to two-thirds of students felt their sleep affected their concentration, productivity or ability to stay awake, and 20% indicated that sleep troubled them in general and for longer than a year. Positive screening rates for probable insomnia were higher in women than men, but the pattern of change across time was similar in the two groups (Fig. 1).

**Table 2:** Description of the Sleep Condition Indicator (SCI-8) items measuring sleep in the past month (percentage (95% CI) reporting) across four successive cohorts of first-year undergraduate students at school entry

	Cohort 1 autumn 2018, pre-pandemic	Cohort 2 autumn 2019, transitional pandemic	Cohort 3 autumn 2020, peak pandemic	Cohort 4 autumn 2021, hybrid pandemic
Time it takes to fall asleep				
0–15 min	34.6 (32.7–36.4)	33.4 (31.5–35.2)	27.5 (24.9–30.1)	30.9 (28.4–33.4)
16–30 min	35.4 (33.5–37.3)	34.6 (32.8–36.5)	31.8 (29.1–34.5)	34.7 (32.2–37.2)
31–45 mins	18.1 (16.5–19.6)	16.8 (15.3–18.3)	19.9 (17.6–22.2)	17.6 (15.6–19.6)
>45 min^a^	12.0 (10.7–13.3)	15.2 (13.8–16.6)	20.8 (18.4–23.1)	16.8 (14.9–18.8)
If waking during the night, awake for…				
0–15 min	66.9 (65.1–68.8)	64.3 (62.4–66.1)	62.4 (59.5–65.2)	63.3 (60.8–65.8)
16–30	18.1 (16.5–19.6)	18.8 (17.3–20.4)	16.2 (14.1–18.4)	18.4 (16.3–20.4)
31–45 min	8.8 (7.7–9.9)	9.3 (8.1–10.4)	9.8 (8.1–11.5)	9.5 (7.9–11.0)
>45 mins^a^	6.2 (5.3–7.2)	7.6 (6.6–8.6)	11.6 (9.8–13.4)	8.9 (7.4–10.3)
Problems with sleep, nights per week				
0–2	68.9 (67.2–70.8)	66.5 (64.7–68.4)	63.4 (60.6–66.2)	68.7 (66.3–71.1)
3–4^a^	21.8 (20.1–23.4)	21.7 (20.1–23.4)	22.0 (19.6–24.4)	19.9 (17.8–21.9)
5–7	9.3 (8.1–10.4)	11.7 (10.5–13.0)	14.6 (12.5–16.6)	11.4 (9.8–13.1)
Sleep quality				
Very good or good	41.0 (39.1–43.0)	40.1 (38.2–42.0)	37.3 (34.5–40.1)	37.2 (34.6–39.8)
Average	41.8 (39.9–43.8)	41.2 (39.3–43.1)	41.4 (38.5–44.2)	44.3 (41.6–46.9)
Poor or very poor^a^	17.1 (15.6–18.6)	18.7 (17.2–20.2)	21.4 (19.0–23.7)	18.5 (16.5–20.6)
Duration of sleep problem				
Do not have a problem or <1 month	60.2 (58.3–62.1)	54.2 (52.2–56.1)	52.9 (50.0–55.8)	55.3 (52.7–57.9)
1–2 months	11.5 (10.3–12.8)	14.3 (12.9–15.7)	11.8 (9.9–13.7)	12.1 (10.4–13.9)
3–12 months^a^	8.5 (7.4–9.6)	8.0 (6.9–9.0)	11.7 (9.8–13.5)	8.4 (6.9–9.9)
>1 year	19.7 (18.2–21.3)	23.5 (21.9–25.2)	23.6 (21.2–26.1)	24.2 (22.0–26.4)
To what extent has your sleep…				
Affected your mood, energy or relationships				
A little or not at all	64.0 (62.1–65.9)	51.9 (49.9–53.8)	58.2 (55.4–61.1)	55.3 (52.7–58.0)
Somewhat^a^	21.4 (19.7–23.0)	25.7 (24.0–27.4)	23.0 (20.5–25.4)	25.7 (23.4–28.0)
Much or very much	14.7 (13.3–16.1)	22.4 (20.8–24.0)	18.8 (16.6–21.0)	19.0 (16.9–21.0)
Affected your concentration, productivity or ability to stay awake				
A little or not at all	46.4 (44.5–48.4)	34.4 (32.5–36.2)	41.3 (38.4–44.1)	35.5 (33.0–38.1)
Somewhat^a^	24.2 (22.5–25.9)	26.9 (25.2–28.6)	24.7 (22.2–27.2)	25.3 (23.0–27.6)
Much or very much	29.4 (27.6–31.2)	38.8 (36.9–40.7)	34.0 (31.3–36.8)	39.2 (36.6–41.8)
Troubled you in general				
A little or not at all	63.8 (61.9–65.7)	51.8 (49.8–53.7)	56.8 (53.9–59.6)	52.8 (50.2–55.5)
Somewhat^a^	20.0 (18.4–21.6)	25.1 (23.4–26.8)	23.3 (20.8–25.7)	25.0 (22.7–27.3)
Much or very much	16.2 (14.7–17.6)	23.1 (21.5–24.8)	20.0 (17.7–22.3)	22.2 (20.0–24.3)
Symptoms of insomnia (SCI-8 ≤ 16)	18.1 (16.6–19.6)	24.1 (22.4–25.7)	29.7 (27.1–32.4)	27.9 (25.6–30.3)

Values for cohorts 1, 3 and 4 are weighted to cohort 2 using propensity scores (inverse probability of treatment weights), developed using age, gender, personal and family history of a mental disorder, international status, parental education and childhood adversities. Some response options were collapsed because of small cell sizes.

a. Cut-off used for identifying a sleep difficulty on the individual item (indicated category or higher equals sleep difficulty).

The multivariable regression results provide further evidence that sleep difficulties and rates of probable insomnia reported at school entry increased before the pandemic (i.e. were more common in autumn 2019 compared with autumn 2018), and then increased further during the peak of the pandemic (autumn 2020) (Supplementary Table 1). Sleep improved somewhat as the university shifted back to in-person teaching (autumn 2021). Students starting in the autumn of 2019 were 31% more likely to screen positive for probable insomnia than those starting the previous year. Similarly, students entering university in the autumn of 2020 (peak pandemic) were 23% more likely to report significant symptoms than those in the autumn 2019 (pre-pandemic) cohort. In contrast, students entering university in the autumn of 2021 were 7% less likely to report significant symptoms than the previous 2020 cohort.

### Changes in sleep over the academic year and the pandemic

**Fig. 2:**
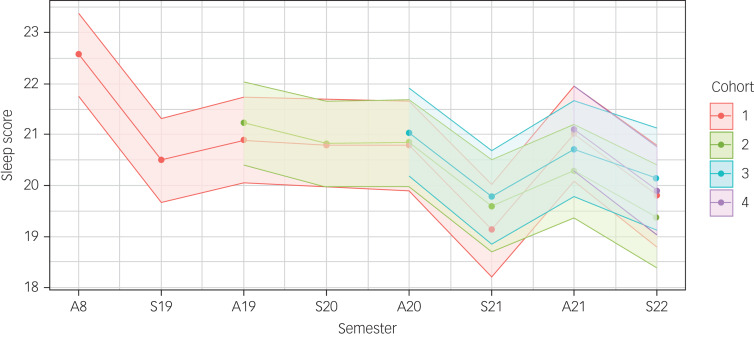
Plot of adjusted estimated mean SCI-8 total scores (95% confidence intervals) over semesters and academic years, by cohort. Linear mixed-effects model adjusted for differences in age, gender, international status, ethnicity, programme of study and parental education level. A18 refers to the 2018 autumn semester, S19 refers to the 2019 spring semester, and so on. Sleep scores are from the SCI-8 (range 0–32), where a higher score indicates better sleep. SCI-8, Sleep Condition Indicator.

The average total SCI-8 score (range 0–32) is plotted in Fig. 2 and described by semester and academic year, adjusting for key covariates and cohort membership in Supplementary Table 2. Sleep was better on average at the start of the academic year (autumn) compared with the end of the academic year (spring) (total SCI-8 scores of 21.1 (95% CI 20.3–21.8) and 20.0 (95% CI 19.2–20.8), respectively). On average, sleep scores decreased, indicating worse sleep, from the 2018–2019 pre-pandemic year to the 2020–2021 peak pandemic year (from 21.5 (95% CI 20.8–22.3) to 20.2 (95% CI 19.4–21.0); average change −0.42 per year; *P*-trend < 0.001). Estimated differences in sleep scores across different semesters and academic years accounting for differences associated with other key covariates are presented in Table 3. The average decrease in sleep scores (indicating worsening sleep) from the autumn to spring semesters was −1.07 (95% CI −1.21 to −0.92). Although statistically significant, this effect is considered fairly small given the pooled s.d. of 8.39.^21^ The greatest decrease in sleep scores over the academic year was seen during the 2018–2019 pre-pandemic year (−2.06; 95% CI −2.41 to −1.71), followed by the 2020–2021 peak pandemic year (−1.39; 95% CI −1.65 to −1.12). Estimated differences in average sleep between academic years provided evidence of a successive worsening of sleep reaching the lowest point during the peak of the pandemic (2020–2021), with some improvement when classes resumed in person and restrictions eased (2021–2022).

**Table 3:** Estimated differences in sleep (Sleep Condition Indicator (SCI-8) total score) over semesters and academic years

Time period contrast	Change in sleep (95% CI)	
Autumn to spring semester (average change)	−1.07	(−1.21 to −0.92)
Autumn 2018 to spring 2019, pre-pandemic	−2.06	(−2.41 to −1.71)
Autumn 2019 to spring 2020, transitional	−0.26	(−0.53 to 0.02)
Autumn 2020 to spring 2021, peak pandemic	−1.39	(−1.65 to −1.12)
Autumn 2021 to spring 2022, hybrid pandemic	−0.98	(−1.22 to −0.74)
Academic year to academic year		
2018–2019 to 2019–2020, pre-pandemic to transitional	−0.61	(−0.88 to −0.35)
2019–2020 to 2020–2021, transitional to peak pandemic	−0.73	(−0.96 to −0.50)
2020–2021 to 2021–2022, peak pandemic to hybrid pandemic	0.10	(−0.11 to 0.30)

Mean differences in total scores on the SCI-8 (range 0–32; lower SCI-8 score indicates worse sleep) estimated using all available data at each time period, using the full linear mixed-methods model adjusting for age, gender, international status, ethnicity, academic programme, highest level of parental education and cohort membership.

### Demographic and lifestyle factors associated with probable insomnia

Evidence from the most recent 2021–2022 academic year (across cohorts) supported that being younger and self-identifying as other than male were associated with screening positive for probable insomnia at the end of the year (spring semester) (Table 4). Further, more frequent cannabis use, spending more time engaged in passive screen time and spending less time on exercise or recreational activities at the start of the academic year were associated with screening positive for probable insomnia at the end of the year. Further, screening positive for insomnia at school entry was significantly associated with screening positive at the end of the academic year (relative risk 1.82; 95% CI 1.74–1.91), and once this was controlled for, effects with other lifestyle variables became more modest.

**Table 4:** Association between demographic and lifestyle variables reported at the beginning of the 2021 academic year and risk of screening positive for probable insomnia at the end of the academic year (spring 2022)

	Bivariate		Adjusted model 1^a^		Adjusted model 2^b^	
	Relative risk	(95% CI)	Relative risk	(95% CI)	Relative risk	(95% CI)
Age, years						
Younger than 18	1.00	Reference	1.00	Reference	1.00	Reference
18 to 19	0.95	(0.89–1.02)	0.95	(0.88–1.01)	0.97	(0.91–1.03)
20 or older	0.96	(0.91–1.02)	0.98	(0.91–1.05)	0.97	(0.91–1.03)
Gender						
Male	1.00	Reference	1.00	Reference	1.00	Reference
Female	1.07	(1.01–1.14)	1.07	(1.01–1.14)	1.02	(0.96–1.08)
Non-binary	1.36	(1.17–1.58)	1.31	(1.14–1.51)	1.20	(1.07–1.35)
Student status						
Domestic	1.00	Reference	1.00	Reference	1.00	Reference
International	0.98	(0.88–1.08)	0.99	(0.89–1.10)	1.03	(0.93–1.14)
Cohort (year of study)						
Cohort 1 (year 4)	1.00	Reference	1.00	Reference	1.00	Reference
Cohort 2 (year 3)	1.08	(0.98–1.18)	1.08	(0.98–1.18)	1.04	(0.96–1.12)
Cohort 3 (year 2)	1.03	(0.95–1.12)	1.06	(0.97–1.16)	1.00	(0.92–1.09)
Cohort 4 (year 1)	1.05	(0.98–1.12)	1.06	(0.97–1.17)	1.01	(0.94–1.09)
Binge drinking						
Never	1.00	Reference	1.00	Reference	1.00	Reference
Monthly or less often	1.01	(0.95–1.07)	1.00	(0.94–1.07)	1.00	(0.94–1.07)
Weekly or more often	1.02	(0.94–1.10)	0.99	(0.91–1.08)	0.98	(0.91–1.06)
Cannabis use						
Never	1.00	Reference	1.00	Reference	1.00	Reference
3 days a month or less	1.08	(1.03–1.13)	1.09	(1.04–1.14)	1.06	(1.01–1.10)
1–2 days a week or more	1.14	(1.06–1.22)	1.15	(1.07–1.24)	1.07	(1.00–1.15)
Exercise, past month						
Never	1.00	Reference	1.00	Reference	1.00	Reference
Once a week or less	0.98	(0.94–1.04)	1.00	(0.95–1.05)	1.02	(0.98–1.06)
2–3 times a week or more	0.88	(0.83–0.93)	0.93	(0.88–0.98)	0.96	(0.92–1.00)
Time for self-care and recreation						
Less than weekly/never	1.00	Reference	1.00	Reference	1.00	Reference
Once a week	0.86	(0.80–0.93)	0.87	(0.81–0.94)	0.93	(0.86–1.01)
2 or more times a week	0.82	(0.76–0.89)	0.84	(0.78–0.90)	0.94	(0.88–1.01)
Passive screen time, h/day						
3 h or less	1.00	Reference	1.00	Reference	1.00	Reference
4–6 h/day	1.04	(0.98–1.10)	1.04	(0.98–1.11)	1.01	(0.95–1.07)
>6 h/day	1.13	(1.04–1.23)	1.12	(1.03–1.22)	1.04	(0.96–1.12)
Screen positive at baseline (SCI-8 ≤ 16)						
No	1.00	Reference	/	/	1.00	Reference
Yes	1.87	(1.77–1.96)	/	/	1.82	(1.74–1.91)

Multiple imputation used to impute missing data. Includes data from all four cohorts (cohort 1 = 517, cohort 2 = 417, cohort 3 = 509 and cohort 4 = 1991). SCI-8, Sleep Condition Indicator.

a. Relative risk adjusted for other variables in the table.

b. Adjusted for other variables in the table, and baseline sleep quality.

## Discussion

In this large ongoing study of university students, sleep difficulties and clinically significant symptoms of insomnia were commonly reported by undergraduate students at entry to university. About a fifth of students reported a sleep problem lasting over a year, and screening positive for insomnia at the beginning of the academic year was strongly associated with screening positive at the end of the year, indicating the persistence of sleep difficulties. Sleep difficulties were reportedly associated with symptoms of cognitive impairment and negatively affected mood, energy and relationships. Probable insomnia was associated with several lifestyle factors, including increased cannabis use and passive screen time, and reduced time spent in exercise, recreation and self-care. With respect to the COVID-19 pandemic, sleep difficulties such as time to fall asleep, poor sleep quality and symptoms of probable insomnia showed a progressive increase from before the pandemic (autumn 2018) to the peak of the pandemic (autumn 2020), when learning was fully remote and pandemic-related restrictions most severe. Sleep improved somewhat when in-person classes resumed and restrictions were eased, but remained below pre-pandemic levels.

Study findings build on the evidence base establishing that sleep problems are common in university students, with an estimated prevalence of insomnia of 18.5% (95% CI 11.2–28.8%) before the pandemic.^22^ Our estimates fall within this range, and are therefore consistent with previous literature. Although sleep problems were most common at the peak of the pandemic, this was an emergent problem that existed before the pandemic, consistent with findings of others,^23,24^ with increases in technology use (smart phones and social media) implicated in the aetiology. Late-night technology use has been associated with night-time awakenings, difficulties getting back to sleep and insomnia.^25^

Based on the U-Flourish survey data, we have demonstrated that psychological risk factors at entry to university (i.e. low self-esteem, external locus of control and negative self-evaluation) are associated with insomnia and anxiety and depressive symptoms at the end of first year. Our observed pattern of an increased prevalence of probable insomnia and sleep difficulties over the pandemic aligns with a recent meta-analysis that reported a pooled prevalence of sleep problems in the general population of 32.3% (95% CI 25.3–40.2%) during the COVID-19 pandemic.^26^ In studies that used the Pittsburgh Sleep Quality Index, this prevalence was estimated at 37.9% (95% CI 25.2–52.4%), and younger age was associated with a higher prevalence of reported sleep problems.

In our analysis, there was a worsening of sleep difficulties over time until the peak of the pandemic. Such trends were associated with moving exclusively to an online learning format, along with other public health restrictions that curtailed living and work situations, social interactions, and sport and recreational pursuits. Pandemic conditions and associated limitations were associated with significant student concern around finances, studies, and the health and wellness of family members and friends.^27^ University students, especially women, reported sleeping more often as a means of coping with the stress of the COVID-19 pandemic.^28^ A majority of university students in the USA (85%) reported changing their sleep habits during the pandemic.^29^ In French university students, inactivity and idleness and academic worries during the pandemic were strongly associated with sleep problems.^30^ In university students in the USA, overall sleep quality did not change with the onset of the pandemic, but they reported greater sleep latency (time to fall asleep) and sleep medication use, and poorer sleep efficiency, associated with the COVID-19 pandemic.^31^ Bedtime and wake time were reported to be significantly later, and sleep duration was significantly longer.^31^ In addition to increased stress and worry, and greater inactivity, the lack of routine, use of asynchronous lectures and time changes for students studying from overseas or in other parts of the country may have contributed to the pandemic-associated rise in sleep problems.

We observed a pattern of worsening sleep from the beginning to end of each academic year across all cohorts, the actual magnitude of which varied from year to year and by phase of the pandemic. This finding aligns with a reported increase in screen-positive rates of anxiety and depressive symptoms over the academic year in undergraduates.^4^ A contributing factor underlying these trends may be increasing levels of stress as academic pressures and responsibilities mount, and final examinations draw closer.^32^ Examination-related anxiety can manifest in many ways, including disrupted sleep and increased fatigue.^33^ Seasonal effects may also play a role, affecting sleep duration, bedtime and wake time.^34^ Specifically, spring has been associated with negative effects on sleep duration, later bedtimes and earlier waking times, possibly related to day length and temperature.

In this study, cannabis use was modestly associated with a higher risk of probable insomnia. An extensive literature supports that substance misuse negatively affects the mental health of young people,^35^ and is associated with a number of negative academic, cognitive and personal outcomes.^36^ In particular, substance use may impede cognitive development, leading to lower academic achievement and performance. Over the course of the COVID-19 pandemic, our data show a decline of alcohol binging in university students, likely reflecting less opportunity for social gatherings. Yet, the pandemic seems to have had a relatively neutral or negative effect on student cannabis use.^7^ The pandemic and associated restrictions severely curtailed recreation and exercise opportunities, while promoting time spent alone and passively on screens and social media.^37^ Striking a healthy study–life balance through investing time in recreational activities and exercise, and reducing passive screen time, has proven benefits for student well-being, sense of connectedness and mental health.^38,39^

Strengths and limitations of this study warrant comment. This study addressed a health priority by using a robust cohort study design. The U-Flourish survey protocol relies on well-validated measures applied in four successively recruited samples of first-year students. Response rates were high compared with other health surveys among college and university students,^40^ especially before the pandemic. Statistical approaches were advanced and adjusted for potential confounding, differential response rates, missing data and attrition. Limitations included an over-representation of women, who report more sleep problems on average than men, leading to the potential for inflated prevalence estimates. Although steps were taken analytically to address potential bias, waning response rates over different stages of the pandemic and points of follow-up may have led to selection bias owing to attrition. Incomplete control for confounders may also be possible. Finally, experiences of Queen's University students may not be generalisable to other student populations, both within and outside of Canada.

Study findings are informative for prevention and the planning of clinical services. First, they highlight that sleep hygiene remains an important focus for health promotion efforts as students transition to university. Second, given that lifestyle affects sleep, student health promotion and mental health literacy efforts should address the negative effects of recreational drug use, passive screen time and reduced exercise on mental health, sleep and academic performance. Third, timely recognition of clinically significant and persistent sleep problems is an important consideration for early intervention efforts, and may unmask associated common mental health concerns such as anxiety and depression. Finally, there appears to be a persistent temporal pattern that predated the COVID-19 pandemic and was exacerbated by it, suggesting that sleep difficulties are becoming endemic in university student populations. The origin, consequences and potential treatment of sleep problems remain a clear priority.

## Supporting information

King et al. supplementary material King et al. supplementary material

## Data Availability

Access to the data and analytic code that support the findings of this study will be considered upon reasonable request to the principal investigator, A.D.
