# Supplementary material for: Changes in sleep and the prevalence of probable insomnia in undergraduate university students over the course of the COVID-19 pandemic: findings from the U-Flourish cohort study
Source: BJPsych Open. 2023 Nov 7;9(6):e210. doi: 10.1192/bjo.2023.597 (PMC10753952; doi:10.1192/bjo.2023.597)
Supplement: King et al. supplementary material [file S2056472423005975sup001.docx]

**Supplementary Figure 1**. Description of cohort sample size and response rates by survey


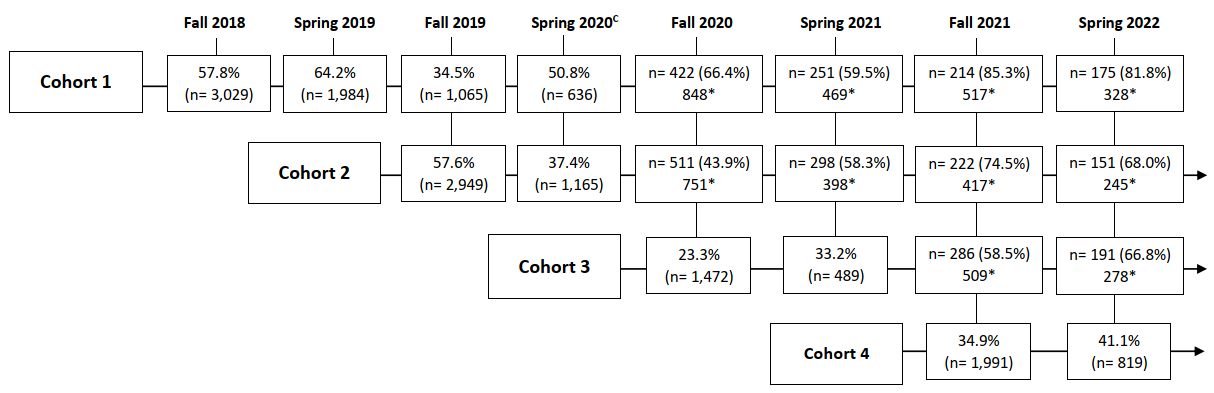


* Includes students that previously dropped out, but came back into the study

^c^ Survey administered shortly after the beginning of the COVID-19 pandemic


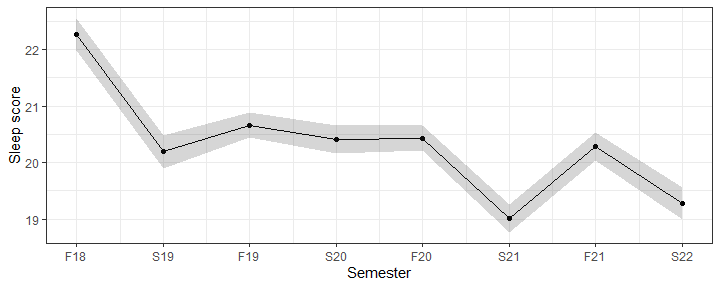


**Supplementary Figure 2**. Plot of estimated mean sleep scores over time (combining all cohorts), from the minimally adjusted LMM.

Legend: F18 refers to the Fall Semester in 2018, S19 refers to the Spring Semester in 2019, etc. Sleep Score refers to the total score on the SCI-8 where a higher score indicates better sleep.

| **Supplementary Table 1.** Multivariable log-binomial regression examining the association between cohort membership and sleep difficulties and screen positive rates reported at school entry, comparing each cohort to the preceding one | | | | | | | |
| --- | --- | --- | --- | --- | --- | --- | --- |
|  | | **Cohort 2 vs Cohort 1**  Pre-pandemic vs  Pre-pandemic | | **Cohort 3 vs Cohort 2**  Peak Pandemic vs.  Pre-pandemic | | **Cohort 4 vs Cohort 3**  Hybrid Pandemic vs. Peak Pandemic | |
| **Sleep Problems** | | **RR** | **(95% CI)** | **RR** | **(95% CI)** | **RR** | **(95% CI)** |
| Screen positives for probable insomnia (SCI≤16) | | 1.31 | (1.16-1.48) | 1.23 | (1.07-1.40) | 0.93 | (0.81-1.06) |
| Had a sleep problem, ≥3 months | | 1.14 | (1.05-1.24) | 1.02 | (0.92-1.13) | 0.94 | (0.85-1.06) |
| Time it takes to fall asleep, *>45 mins* | | 1.06 | (0.96-1.17) | 1.26 | (1.13-1.42) | 0.84 | (0.75-0.96) |
| If wake up during the night, *awake >45 mins* | | 1.11 | (0.96-1.27) | 1.25 | (1.07-1.46) | 0.83 | (0.70-0.98) |
| Nights/week have a problem with sleep, *≥3 nights/week* | | 1.24 | (1.05-1.48) | 1.23 | (1.02-1.48) | 0.80 | (0.65-0.98) |
| *Sleep quality, poor or very poor* | | 1.06 | (0.93-1.21) | 1.13 | (0.97-1.32) | 0.87 | (0.74-1.03) |
| *Sleep has somewhat to very much…* | |  |  |  |  |  |  |
|  | Affected your mood, energy or relationships | 1.50 | (1.31-1.71) | 0.85 | (0.73-0.99) | 0.96 | (0.81-1.14) |
|  | Affected your concentration, productivity, or ability to stay awake | 1.31 | (1.19-1.44) | 0.89 | (0.79-1.00) | 1.09 | (0.96-1.24) |
|  | Troubled you in general | 1.41 | (1.24-1.60) | 0.87 | (0.75-1.02) | 1.06 | (0.90-1.25) |
| Note: Used all available data from each cohort (Fall 2018 (Cohort 1)= 2501, Fall 2019 (Cohort 2)= 2575, Fall 2020 (Cohort 3)= 1201, Fall 2021 (Cohort 4)= 1500), and adjusted for age, gender, personal and family history of a mental disorder, international status, parental education, and childhood adversities. | | | | | | | |

| **Supplementary Table 2**. Marginal mean sleep as measured by the total score on the SCI-8 (higher scores= better sleep) of undergraduate students by semester and academic year | | | |
| --- | --- | --- | --- |
| **Contrast** | **Mean** | **SE** | **(95% CI)** |
| Fall Semesters | 21.05 | 0.392 | (20.28-21.82) |
| Spring Semesters | 19.98 | 0.398 | (19.20-20.76) |
| 2018/2019 Academic Year: Pre-pandemic | 21.54 | 0.405 | (20.75-22.34) |
| 2019/2020 Academic Year: Transitional | 20.93 | 0.399 | (20.15-21.71) |
| 2020/2021 Academic Year: Peak Pandemic | 20.19 | 0.400 | (19.41-20.98) |
| 2021/2022 Academic Year: Hybrid Pandemic | 20.29 | 0.402 | (19.50-21.08) |
| Note: Mean total SCI scores (Range 0-32) estimated using the full LMM model adjusting for age, gender, international status, ethnicity, academic program, highest level of parental education, and cohort membership (year of study). | | | |
